# Gene Flow and Vertical Stratification of Pollination in the Bat‐Pollinated Liana *Marcgravia longifolia*

**DOI:** 10.1002/ece3.72050

**Published:** 2025-08-23

**Authors:** Malika Gottstein, Sarina Thiel, Jan Lukas Vornhagen, Christina Mengel, Marco Tschapka, Eckhard W. Heymann, Katrin Heer

**Affiliations:** ^1^ Eva Mayr‐Stihl Professorship for Forest Genetics, Faculty of Environment and Natural Resources, Albert‐Ludwigs‐Universität Freiburg Freiburg im Breisgau Germany; ^2^ Museum für Naturkunde—Leibniz Institute for Evolution and Biodiversity Science Berlin Germany; ^3^ Department of Biology, Conservation Ecology Philipps‐Universität Marburg Germany; ^4^ Institute of Evolutionary Ecology and Conservation Genomics University of Ulm Ulm Germany; ^5^ Smithsonian Tropical Research Institute Ancon Republic of Panama; ^6^ Verhaltensökologie & Soziobiologie Deutsches Primatenzentrum—Leibniz‐Institut für Primatenforschung Göttingen Germany

**Keywords:** Amazon rainforest, chiropterophily, forest strata, pollen dispersal, pollinator‐mediated gene flow, spatial genetic structure, dispersión de polen, estratos forestales, estructura genética espacial, flujo génico mediado por polinizadores, selva amazónica, quiropterofilia

## Abstract

Pollen dispersal is a key driver of gene flow in plant populations, shaping their spatial genetic structure (SGS). In tropical forests, plant‐pollinator interactions vary across vertical strata due to differences in microclimate, resource availability, and foraging behavior. Bats are an important tropical pollinator group and have been observed to exhibit vertical stratification in their foraging activity, with interaction frequencies differing across forest layers. They are highly mobile and expected to transport pollen over long distances; however, their actual contribution to gene flow has rarely been investigated. *Marcgravia longifolia*, a bat‐pollinated Neotropical liana, offers a unique system for studying gene flow across forest strata. Unlike most other plant species, *M. longifolia* produces flowers from the forest floor to the canopy, allowing us to study how bat pollination differs across strata. This study examines pollen dispersal distances, the vertical stratification of gene flow, and SGS in *M. longifolia* at a 100 ha study site in western Amazonia. Pollen dispersal distances were up to 1350 m, with longer distances observed in the understory and midstory, where bat foraging activity is more frequent. We detected no SGS, suggesting extensive gene flow facilitated by bat pollination across forest strata. These findings underscore the critical role of bats in shaping plant genetic structure and demonstrate how vertical forest stratification influences gene flow in tropical ecosystems.

## Graphical abstract

This study investigates pollen dispersal, vertical stratification of gene flow, and spatial genetic structure in the bat‐pollinated liana *Marcgravia longifolia* in a western Amazonian forest. Flowers are produced from the forest floor to the canopy, allowing analysis of pollination across strata. Results show that bats disperse pollen up to 1350 m, especially in lower forest layers, and facilitate extensive gene flow, highlighting their role in maintaining genetic connectivity in vertically structured tropical ecosystems.

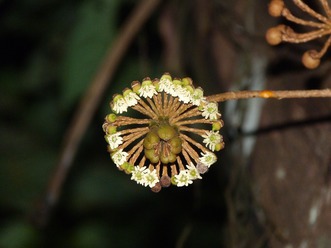

## RESUMEN

La dispersión del polen es un factor clave del flujo génico en las poblaciones de plantas, dando forma a su estructura genética espacial (SGS). En los bosques tropicales, las interacciones planta‐polinizador varían a lo largo de los estratos verticales debido a diferencias en el microclima, la disponibilidad de recursos y el comportamiento de forrajeo. Los murciélagos son un grupo de polinizadores importante en los trópicos y se ha observado que presentan una estratificación vertical en su actividad de forrajeo, con frecuencias de interacción que difieren entre los distintos estratos del bosque. Son altamente móviles y se espera que transporten polen a largas distancias; aunque su contribución real al flujo génico ha sido raramente investigada. *Marcgravia longifolia*, una liana neotropical polinizada por murciélagos, ofrece un sistema único para estudiar el flujo génico a través de los estratos del bosque. A diferencia de la mayoría de las especies de plantas, *M. longifolia* produce flores desde el suelo del bosque hasta el dosel, lo que permite estudiar cómo varía la polinización por murciélagos en los distintos niveles. Este estudio examina las distancias de dispersión del polen, la estratificación vertical del flujo génico y la SGS en *M. longifolia* en un sitio de estudio de 100 hectáreas en la Amazonía occidental. Las distancias de dispersión del polen alcanzaron hasta 1350 metros, observándose distancias mayores en el sotobosque y el estrato medio, donde la actividad de forrajeo de los murciélagos es más frecuente. No se detectó SGS, lo que sugiere un flujo génico extenso facilitado por la polinización por murciélagos a través de los estratos del bosque. Estos resultados destacan el papel crucial de los murciélagos en la formación de la estructura genética de las plantas y demuestran cómo la estratificación vertical de los bosques influye en el flujo génico en los ecosistemas tropicales.

## 1 Introduction

Gene flow, primarily facilitated through pollen and seed dispersal, plays a fundamental role in maintaining genetic diversity within seed plant populations (Browne et al. 2018; Campbell 1991; Dick et al. 2008). In tropical ecosystems, where over 95% of flowering plant species rely on animal pollinators, biotic vectors such as insects, birds, and mammals are essential to the reproductive success of many plant species (Bawa 1990; Ollerton et al. 2011). Among these vectors, bats have emerged as particularly effective pollinators in tropical landscapes, offering distinct advantages for plants. Compared to other pollinators, bats transport larger amounts of pollen, due to their generally larger body size relative to insects and due to the structure of their fur, which retains more pollen than the feathers of hummingbirds of similar size (Moreira‐Hernández et al. 2021; Muchhala and Thomson 2010). Also, pollen transfer is more targeted, as bats visit a smaller number of species, and they tend to deposit a high diversity and quantity of pollen (Diniz et al. 2019; Fleming et al. 2009; Tschapka and Dressler 2002). Additionally, plants may reward bats with less nectar than non‐flying mammals with a similar mobility range (Tschapka and von Helversen 1999; von Helversen et al. 2003). While many nectarivorous bats are small‐bodied and occupy small home ranges, thus producing short pollination distances (Heithaus et al. 1975; Rothenwöhrer et al. 2011), the ecological roles and movement capacities of different species vary widely and are influenced by habitat structure and landscape composition (Biscaia de Lacerda et al. 2008; Collevatti et al. 2010; Diniz et al. 2019; Klingbeil and Willig 2009). Bats occupy specific ecological niches within tropical forests, often exhibiting pronounced vertical stratification across forest strata. While some bat species primarily forage in the understory, others are specialized to forage almost exclusively in the canopy (Bernard 2001; Gregorin et al. 2017; Kalko and Handley 2001; Rex et al. 2011; Thiel et al. 2021). This vertical stratification plays a critical role in structuring biodiversity within tropical forests and significantly influences ecological processes such as pollination (Thiel et al. 2021, 2024).

**FIGURE 1:**
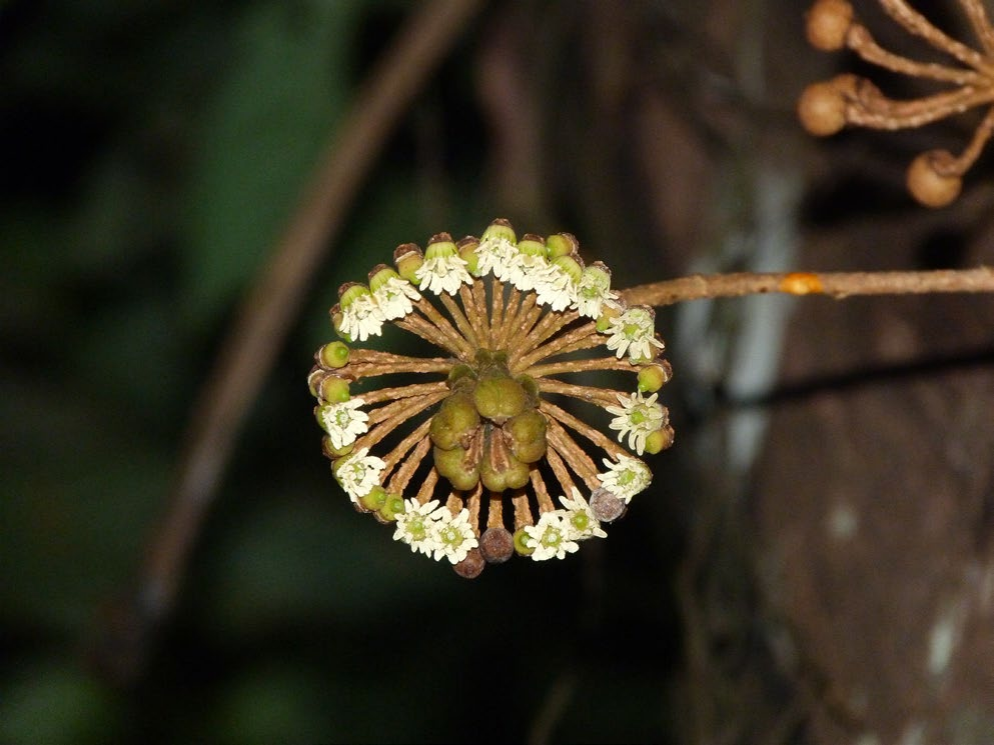
Inflorescence of *Marcgravia longifolia* blooming at night. Photo by Eckhard W. Heymann.

*Marcgravia longifolia* J.F.Macbr. (1934) is a woody liana species distributed in western Amazonia (Dressler 2004; Tropicos 2024). It offers a unique model for studying gene flow across forest strata due to its flowering and fruiting along the entire vertical gradient from the understory to the canopy. This species produces flagelliflorous inflorescences on the unbranched stem, all the way from ground level up to the canopy. *Marcgravia longifolia* is pollinated by nectarivorous bats. In a previous study on *M. longifolia* at our study site, Thiel et al. (2024) found bats to visit *M. longifolia* flowers across all strata, with the highest visitation rates in the midstory and the lowest in the canopy. Observed pollinators were *Hsunycteris thomasi* , *Choeroniscus minor* , and *Anoura caudifer* (Thiel et al. 2024). The inflorescences show characteristic adaptations to chiropterophily, such as blooming at night, whitish coloration, and large nectaries (Fleming et al. 2009; Tschapka and Dressler 2002; Vogel 1958). Each inflorescence consists of 20–25 flowers arranged in a circle around three to eight extrafloral saccate nectaries (Tirado Herrera et al. 2003; Figure 1). Flowers open between 6 and 7 pm by shedding the cap‐like fused petals and revealing a ring of white stamen (Tirado Herrera et al. 2003). The anthesis of inflorescences lasts for three consecutive nights (Willems 2016). Nectarivorous bats visit *M. longifolia* flowers mostly in the under‐ and midstory, driven by the higher abundance of inflorescences in these strata (Thiel et al. 2024). The flowering period in *M. longifolia* is 1.42 months on average, with a peak in August (Mainardi et al., n.d., submitted). Seeds of *M. longifolia* are dispersed by birds and primates (Thiel et al. 2023; Tirado Herrera et al. 2003).

In this study, we investigate gene flow in *M. longifolia* by assessing pollen dispersal distances, vertical stratification of pollen dispersal, and the spatial genetic structure (SGS). We hypothesize that (1) bats move pollen across the entirety of our study area; (2) gene flow is higher in the under‐ and midstory in *M. longifolia* , where foraging by nectar‐feeding bats is more frequent; and (3) SGS is minimal, given the high dispersal distances expected from diverse pollen (and seed) dispersal vectors. Through this research, we aim to deepen our understanding of the ecological processes that drive gene flow in bat‐pollinated tropical plants and clarify how forest stratification influences genetic dynamics within species.

## 2 Materials and Methods

### 2.1 Study Site

We collected the genetic samples used in this study at the Estación Biológica Quebrada Blanco (EBQB) in Peru (4°21′ S 73°09′ W). Mean monthly temperatures range from 25°C to 27°C and annual rainfall in the area is ca. 3000 mm with a clear seasonal pattern (Lüffe et al. 2018; Myster 2015). The EBQB is mostly covered by high ground terra firme rainforest (“bosque de altura”, (Encarnación 1993)) both on flat/slightly undulating or strongly undulating terrain (“bosque de de terraza” and “bosque de colina”, respectively (Encarnación 1993)), interspersed with swampy areas. A more detailed description of the study site can be found in Heymann et al. (2021) and Heymann and Tirado Herrera (2021).

### 2.2 Sample Collection

**FIGURE 2:**
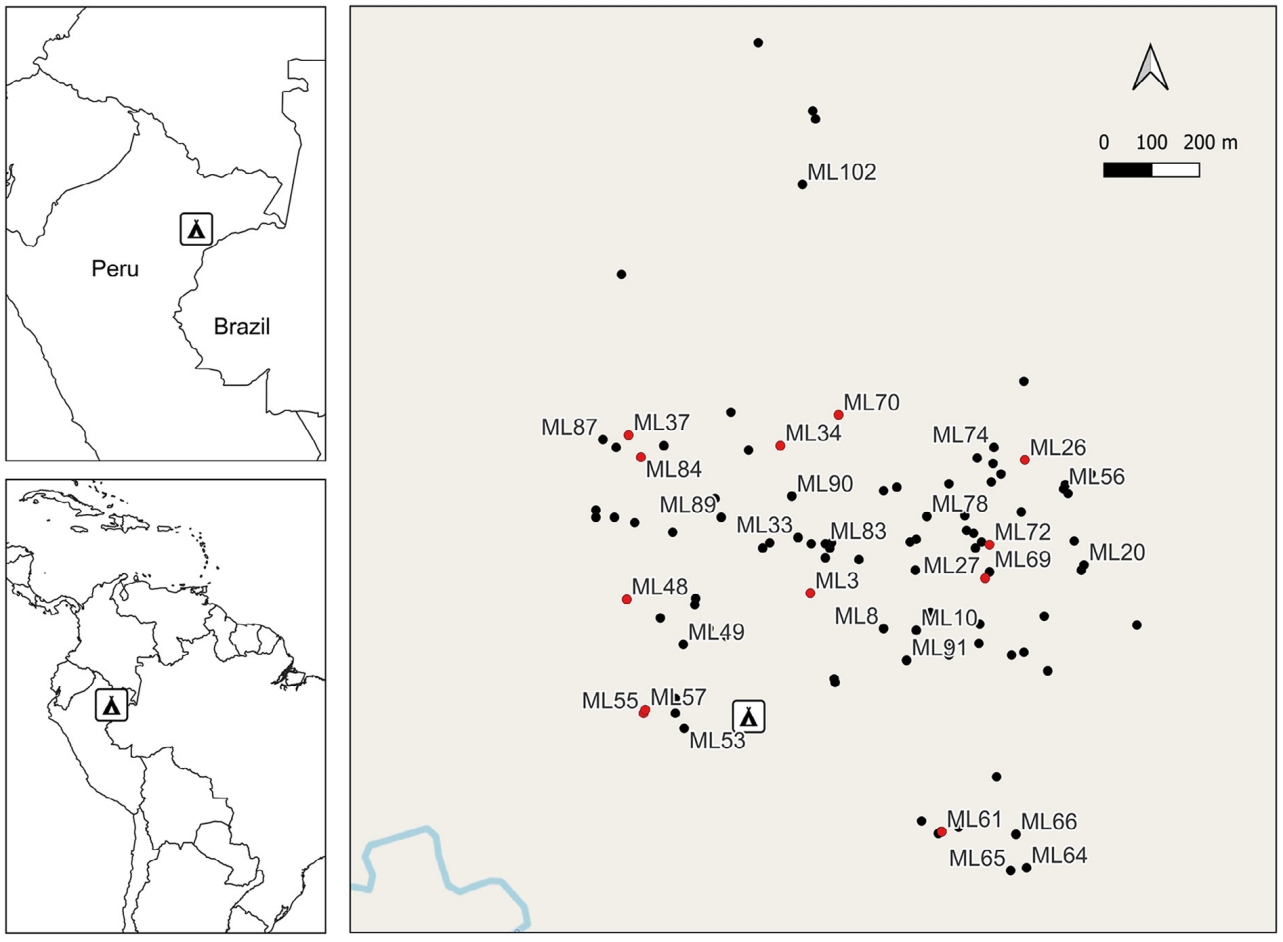
Map of adult *M. longifolia* at our study site. Black dots are individuals from which we collected cambium samples, red dots are individuals from which we collected cambium samples and fruits. Parents are labeled with ID numbers to indicate the location of known mothers and assigned fathers for seeds from Table A1. Map created using QGIS 3.40.5.

Since 2015, we systematically conducted extensive searches for *M. longifolia* in the EBQB area, encompassing all habitats suitable for the species. By 2018, we had located 105 adult *M. longifolia* , mainly growing within the 100 ha (1 km^2^) study area covered by a trail grid (see Heymann et al. 2022) and some in accessible areas outside this grid (Figure 2). All individuals were georeferenced with a Garmin GPSMAP 65s. *Marcgravia longifolia* is easily recognized in the field due to its distinctive growth form, and occurs only on “bosque de terraza” (Heymann et al. 2022). We also conducted exploratory surveys beyond the boundaries of the study site and did not locate any additional *M. longifolia* individuals, suggesting that the species has a low density or is absent in the surrounding matrix, likely due to changes in the forest type. Furthermore, our field searches began in 1997 during earlier ecological studies (Tirado Herrera et al. 2003), and members of our team (NST and EWH) have worked continuously at this site for over 30 years, providing deep familiarity with the landscape. We are confident that we have located all reproductively mature individuals within the EBQB area. While we believe that our surveys also captured the majority of individuals occurring in the immediate surroundings, we cannot be entirely certain that no additional adults exist beyond the EBQB boundaries. We collected cambium samples from all adult *M. longifolia* , except for five individuals that died early during fieldwork. Using a sterilized pocketknife, we scraped the outer layers from a 1 × 1 cm^2^ area of the stem and then abraded the cambium layer. The samples were stored in paper bags on silica gel for up to 6 months before being shipped to the laboratory.

We collected ripe fruits from 13 *M. longifolia* individuals over two periods: October to December 2018 and October to December 2019. In both periods, we sampled 10 of the 13 individuals, resulting in data for both years for seven individuals and data for only one year for six individuals. From each infructescence, we collected one to six ripe fruits, recording the height in meters and the associated forest stratum. We marked the infructescences for resampling.

We categorized the forest into three strata with clearly distinct vegetation density: understory, midstory, and canopy (Thiel et al. 2024). We chose to classify the forest strata based on vegetation structure rather than absolute height, as we assume that nectarivores navigate largely according to structural cues instead of specific heights. Thus, the understory, determined by the height of the surrounding shrub and palm layer, varied in height between 3 and 10 m. The canopy was defined as the space between the first major canopy branch (at 9–12 m) of the host tree and its total height (22–32 m). The midstory encompassed the vertical space between the understory and canopy. A figure showing the height distribution in the sampled *M. longifolia* individuals can be found in Thiel et al. (2024, appendix S3). Using a single‐rope climbing technique, we accessed the infructescences and collected the fruits by hand. In total, we collected 729 fruits (Table 1).

**TABLE 1:** Number of genotyped seeds from *Marcgravia longifolia* by individual, sampling year, and forest stratum.

ID *M. longifolia* individual	Year	Number of genotyped seeds			
		Understory	Midstory	Canopy	Total
ML3	2018	1	6	4	11
ML3	2019	12	4	8	24
ML20	2018	17	12	0	29
ML26	2018	9	3	5	17
ML26	2019	5	8	6	19
ML27	2018	6	3	0	9
ML27	2019	7	8	6	21
ML34	2018	6	6	0	12
ML34	2019	8	4	7	19
ML37	2018	15	6	11	32
ML48	2018	11	16	6	33
ML55	2018	6	7	0	13
ML55	2019	8	16	0	24
ML57	2018	3	7	0	10
ML57	2019	7	14	0	21
ML61	2019	10	10	9	29
ML70	2019	5	11	9	25
ML72	2019	9	11	10	30
ML84	2018	3	6	0	9
ML84	2019	6	6	0	12
Total		154	164	81	399

Since the seeds of *M. longifolia* are tiny (0.06 cm × 0.06 cm × 0.06 cm; Knogge 1999), direct DNA extraction from the seeds was not feasible. We therefore germinated a part of the seeds of each fruit, partially in the field and partially in a greenhouse. After separating the seeds from the fruit pulp, seeds from each fruit were placed in an individually labeled pot. Within 2–3 months, the seeds developed cotyledons, which were then dried and stored on silica gel for up to 12 months. We used all seeds that developed cotyledons for genotyping (*n* = 399, Table 1).

### 2.3 DNA Extraction

We extracted DNA from the dried cotyledons and cambium using a modified ATMAB protocol as described in Dumolin et al. (1995), based on Hewitt et al. (1991). Samples were placed in 2 mL Eppendorf tubes, frozen in liquid nitrogen, and ground with steel beads using a laboratory mill at 25 Hz for 3 min. We then added 1 mL of preheated ATMAB extraction buffer and 50 μL of DTT, and incubated the tubes at 55°C for 1 h, turning them every 15 min. After a 10‐minute cooling period, 400 μL of dichloromethane was added, and the tubes were centrifuged at 4°C and 13,000 rpm for 10 min. The upper phase (~650 μL) was transferred to new 1.5 mL Eppendorf tubes, to which 400 μL of cold isopropanol was added. The tubes were gently swayed to detach the pellet and left overnight to ensure complete DNA precipitation. After a second centrifugation at 4°C and 13,000 rpm for 10 min, the supernatant was removed, and the tubes were left to dry for 5 min. Then 1 mL of cold 76% ethanol was added to wash the pellet, followed by another centrifugation and removal of the supernatant. The tubes were left to dry completely for at least 5 h. Finally, 50 μL of 1× TE + RNase (1 mg/mL) was added for samples from adult plants, and 30 μL for cotyledons. After overnight dissolution, the tubes were incubated at 37°C for 30 min. The resulting DNA stock solution was stored at −20°C and diluted as needed before use.

### 2.4 Microsatellite Development

Microsatellites were developed by ecogenics (2018). First, an Illumina TruSeq Nano library was created from one individual and sequenced using an Illumina MiSeq platform with a Nano v2 500‐cycle sequencing chip. Paired‐end reads that passed Illumina's chastity filter were de‐multiplexed, and Illumina adaptor residuals were trimmed. Quality checks were performed using FastQC v0.117 (Andrews 2018). Paired‐end reads were merged using USEARCH v10.0.240 (Edgar 2010) to reconstruct the sequence. Merged reads were screened with Tandem Repeats Finder v4.09 (Benson 1999), identifying 8215 reads containing a microsatellite insert with a tetra‐ or trinucleotide of at least six repeat units or a dinucleotide of at least ten repeat units. Primer design was performed with Primer3 (Koressaar and Remm 2007; Untergasser et al. 2012), yielding 4177 suitable microsatellite candidates. After testing for amplification efficiency and polymorphism, twelve primers were selected that exhibited high reliability of amplification and polymorphism.

We initially analyzed all samples at twelve microsatellite loci, organized into five multiplexes based on annealing temperature (Table 2). We assigned markers to multiplex reactions as follows: ML373830, ML657826, and ML741518 (Multiplex 1); ML220529 and ML976893 (Multiplex 2); ML246705, ML330921, and ML1311224 (Multiplex 3); ML252171 and ML600839 (Multiplex 4); ML451323 and ML1369117 (Multiplex 5). Primers within the same multiplex were labeled with different dyes. PCR reactions were carried out in a total volume of 14.6 μL, using 2 μL of diluted DNA (10 ng/μL) per reaction. The PCR mix included 10× PCR buffer, MgCl_2_ (concentration optimized per multiplex, column CoF, Table 2), 1 μL of dNTP mix, 0.13 μL of Taq polymerase, 0.13 μL of BSA, and forward and reverse primers (volumes adjusted per primer pair, column P_FR_, Table 2). Amplifications were performed on a Biometra TOne thermocycler (Analytik Jena AG, Jena, Germany) with the following cycling conditions: initial denaturation at 94°C for 5 min, followed by 35 cycles of 94°C for 45 s, locus‐specific annealing temperature (*T*_a_) for 45 s, and 72°C for 45 s, with a final extension at 72°C for 10 min. PCR products were separated using capillary electrophoresis on an ABI 3730 Genetic Analyzer and scored with GeneMapper v5.0 (Applied Biosystems). After evaluating marker performance, we retained six loci for further analysis.

**TABLE 2:** Characteristics of microsatellite markers for *Marcgravia longifolia*.

Primer name	Primer sequences (5′–3′)	Repeat motif	*T*_a_ [°C]	CoF [μL]	P_FR_ [μL]	Multiplex	Dye
ML252171^a^	F: AGTCATTTGCATTCTTAGGCG	(TTTG)_13_	55.5	1.7	0.95	4	Vic
	R: AAATCCAAAACCGAGGTGGC						
ML330921^a^	F: TTAGCCTGTCACTGAGCGTG	(GGA)_8_	57	2	0.85	3	Pet
	R: TGGTTTCGCCTAAGACTGGG						
ML373830^a^	F: CAAGCCGATAATGGCACTGG	(TCT)_11_	58	1.7	0.85	1	Pet
	R: CGTCTTTTCCGTAACTGGAGC						
ML657826^a^	F: TGGATAAGCTTTTGTTGTCGGTC	(GA)_12_	58	1.7	0.85	1	Ned
	R: ATCACACCGTGCAATTAGGC						
ML976893^a^	F: TGCTCGAAAATGGAAGCCATAC	(TCT)_8_	59	1.7	0.95	2	Fam
	R: CCTCTCTGCAATTATGGACGG						
ML1369117^a^	F: TCAAGGACGTGATGTTTTGCC	(AAAG)_7_	57	1.7	0.75	5	Vic
	R: ACGAGGCATGTTCCCTGTG						
ML220529	F: GTTCCTTCTTGCTCACTCTAGC	(TC)_20_	59	1.7	0.85	2	Vic
	R: ACCTTCAACTCCACGCTCTG						
ML246705	F: ATATCTGGGGGAGCGCATAG	(AG)_15_	57	2	0.85	3	Ned
	R: GCTACAATTCAGCGTGGGTG						
ML451323	F: ACCGAGTAATTGCGTCTTCTC	(TAT)_21_	57	1.7	0.95	5	Fam
	R: CATTGCGAGGGTCTACGAAC						
ML600839	F: TTGCTAACAAGTCCATGGGG	(GA)_17_	55.5	1.7	0.95	4	Fam
	R: TGGGCCGTACATCAATCCTC						
ML741518	F: AGTAGAGTCCATAAACTAAGGCG	(CTT)_10_	58	1.7	0.85	1	Fam
	R: TGCTATGTTTCACCCAAGCC						
ML1311224	F: CGATGCCCTCACTAGATCCC	(TCT)_11_	59	1.7	0.85	3	Fam
	R: TCTGAGCATCGAAGTGTACG						

Abbreviations: CoF, co‐factor MgCl_2_; P_FR_, primer (F&R); *T*_a_, annealing temperature.

^a^ Used for paternity assignment.

### 2.5 Genotyping

We genotyped the samples using capillary gel electrophoresis on an ABI Genetic Analyzer 3500 (Thermo Fisher Scientific, Waltham, MA, USA). Depending on the marker, we mixed 1.5, 2, or 3 μL of PCR product with 9.8 μL Hi‐Di Formamide and 0.2 μL GeneScan 600 LIZ dye Size Standard (Thermo Fisher Scientific). Each multiplex solution was transferred into a 96 microwell plate (Kisker Biotech G060/H/1E‐OA). We analyzed the results with GeneMarker 2.7.0 (SoftGenetics 2016) using predefined panels, which we verified to ensure accurate genotyping. Each sample was then reviewed visually to confirm or adjust the scores. If results were unclear, we repeated the PCR and ABI run. After data review, six markers were eliminated due to errors such as allele inconsistencies between known offspring and mothers or irreproducible results.

We assigned paternity by maximum likelihood using Cervus 3.0.7 (Kalinowski et al. 2007, 2010). Cervus analyzes genetic data from co‐dominant genetic markers, such as microsatellites, assuming that the species is diploid, that markers are autosomal and inherited independently of each other (Marshall 2014). We first determined the allele frequencies and summary statistics for adults and seeds for all loci, including expected heterozygosity, polymorphic information content (PIC), exclusion probabilities, Hardy–Weinberg equilibrium, and null allele frequency estimates. To determine critical values for paternity assignment, we conducted simulations in which Cervus generated log‐likelihood ratios (LOD scores) for pairs of candidate parents and offspring. These LOD scores represent the logarithm of the likelihood that a given candidate is the true father relative to a randomly selected unrelated adult individual. The distribution of LOD scores from these simulations was then used to establish confidence thresholds for paternity assignment.

Paternity assignments were made from the pool of successfully genotyped adults, which represent 80% of the adult *M. longifolia* individuals in the study area, since some sampled adults could not be genotyped successfully. A minimum of three loci was required for inclusion in the analysis, as the probability of identity (PID) dropped below 5% with at least three loci. PID refers to the probability that two unrelated individuals will, by chance, share an identical genotype (Marshall 2014). Lower PID values indicate greater discriminatory power of the marker set. These values depend on species‐specific levels of genetic diversity and can vary across loci (Waits et al. 2001).

Paternity was assigned using the relaxed confidence levels of 80% in Cervus, meaning that a candidate parent was considered the likely father only if the simulated probability of correct assignment was at least 80%. This confidence value takes into account the number of candidate parents, the proportion of candidate parents sampled, the completeness of genetic typing, and the estimated frequency of typing errors (Marshall 2014). In Cervus, the confidence value reflects the statistical support for each paternity assignment and is distinct from the paternity exclusion probability, which measures the overall statistical power of the marker set to exclude unrelated adult individuals as potential fathers. We pre‐assigned known mothers based on sampling information. The final dataset included 105 adult *M. longifolia* individuals and 355 seeds, of which 96 adults and 340 seeds were genotyped at a minimum of three loci and included in the analysis. We assigned paternity to 72 seeds, mostly from the understory (*n* = 32), followed by seeds from the midstory (*n* = 24) and canopy (*n* = 16).

### 2.6 Pollen Dispersal

We measured the distance between assigned fathers and known mothers of *M. longifolia* seeds. Each seed was classified according to the forest stratum (understory, midstory, or canopy) from which the fruit was collected from the mother plant. To examine pollen dispersal distances (response variable) in relation to height and stratum (explanatory variables), we used a linear model for height, as the relationship between height and dispersal distance was linear, and the residuals were both homoscedastic and normally distributed. To assess differences in dispersal distances among strata, we applied a Kruskal‐Wallis test, followed by a post hoc Dunn's test. The analyses and visualizations were performed using R v4.2.2, using packages geosphere, geodist, spatstat, and sp. (Baddeley et al. 2002; Hijmans 2010; Padgham and Sumner 2018; Pebesma and Bivand 2005; R Core Team 2022).

### 2.7 Spatial Genetic Structure (SGS)

We used SPAGeDi 1‐5d (Hardy and Vekemans 2002) to characterize the SGS of adult *M. longifolia* individuals at our study site. We assessed the genetic structure using a spatial auto‐correlation analysis of genetic relatedness between the individuals, using the kinship coefficient of Loiselle et al. (1995). Additionally, we computed mean jackknifed estimators and jackknifed standard errors over all loci for kinship coefficients. The number of distance classes used was set to five after assessing the data and with the intent of achieving an even spread of pairwise comparisons between all classes. We computed the Sp value and its *p*‐value in order to assess the intensity of SPS (Vekemans and Hardy 2004). The Sp statistic is defined as Sp = −b/(*F*_1_–1), with b as the slope of the regression of F_ij_ on ln (d_ij_), the natural logarithm of the spatial distance between individuals, and *F*_1_ being the average kinship coefficient between individuals of the first distance class (maximum distance 269 m). We included all adult individuals of *M. longifolia* in the analysis for which the geographic coordinates and the genotype from at least one locus were available (*n* = 97).

## 3 Results

### 3.1 Genetic Diversity and Marker Performance

Across the six microsatellite loci retained for analysis, the number of alleles per locus ranged from 5 to 11 in adult *M. longifolia* individuals (Table 3) and from 5 to 14 in seeds (Table 4). Observed heterozygosity ranged from 0.261 to 0.835 in adults (mean: 0.636) and from 0.286 to 0.626 in seeds (mean: 0.518). Expected heterozygosity was generally higher than observed, with values ranging from 0.382 to 0.794 in adults (mean: 0.654) and from 0.386 to 0.795 in seeds (mean: 0.637). Polymorphic information content (PIC) values were moderately high overall, ranging from 0.361 to 0.760 in adults and 0.365 to 0.769 in seeds. The combined non‐exclusion probabilities for identifying a parent pair were 0.002 for adults and 0.003 for seeds, indicating good discriminatory power for paternity analysis.

**TABLE 3:** Summary statistics of allele frequency analysis for adult *M. longifolia* .

Locus	*N*	HObs	HExp	PIC	NE‐1P	NE‐2P	NE‐PP	NE‐I	NE‐SI	HW	*F*(Null)
ML252171	6	0.761	0.733	0.684	0.681	0.506	0.323	0.118	0.415	NS	−0.0245
ML330921	6	0.593	0.627	0.579	0.780	0.610	0.425	0.187	0.485	NS	0.0308
ML373830	9	0.609	0.672	0.642	0.718	0.530	0.319	0.136	0.450	NS	0.0486
ML657826	11	0.835	0.794	0.760	0.583	0.406	0.221	0.074	0.374	NS	−0.0278
ML976893	5	0.261	0.382	0.361	0.923	0.783	0.633	0.404	0.661	ND	0.1936
ML1369117	7	0.756	0.716	0.674	0.690	0.510	0.318	0.121	0.424	NS	−0.0295
Combined non‐exclusion probability					0.142	0.027	0.002	0.00001	0.009		

Abbreviations: *F*(Null), estimated null allele frequency; HExp, expected heterozygosity; HObs, observed heterozygosity; HWE, deviation from Hardy–Weinberg equilibrium; *N*, number of alleles; NE‐1P, non‐exclusion probability for first parent; NE‐2P, non‐exclusion probability for second parent; NE‐I, non‐exclusion probability for identity; NE‐PP, non‐exclusion probability for parent pair; NE‐SI, non‐exclusion probability for sib identity; PIC, polymorphic information content.

**TABLE 4:** Summary statistics of allele frequency analysis for *M. longifolia* seeds.

Locus	*N*	HObs	HExp	PIC	NE‐1P	NE‐2P	NE‐PP	NE‐I	NE‐SI	HWE	*F*(Null)
ML252171	8	0.617	0.708	0.656	0.709	0.539	0.357	0.137	0.431	*	0.0701
ML330921	10	0.516	0.596	0.546	0.804	0.643	0.464	0.213	0.506	*	0.0766
ML373830	10	0.438	0.628	0.602	0.758	0.571	0.360	0.164	0.478	***	0.1895
ML657826	14	0.626	0.795	0.769	0.566	0.388	0.199	0.068	0.370	***	0.1113
ML976893	7	0.286	0.386	0.365	0.920	0.780	0.627	0.398	0.657	***	0.1347
ML1369117	5	0.622	0.709	0.665	0.702	0.525	0.338	0.128	0.428	NS	0.0654
Combined non‐exclusion probability					0.158	0.031	0.003	0.00002	0.011		

Abbreviations: *F*(Null), estimated null allele frequency; HExp, expected heterozygosity; HObs, observed heterozygosity; HWE, deviation from Hardy–Weinberg equilibrium; *N*, number of alleles; NE‐1P, non‐exclusion probability for first parent; NE‐2P, non‐exclusion probability for second parent; NE‐I, non‐exclusion probability for identity; NE‐PP, non‐exclusion probability for parent pair; NE‐SI, non‐exclusion probability for sib identity; PIC, polymorphic information content.

NS: Not statistically significant, *p* > 0.05; *: *p* < 0.05 and ***: *p* < 0.001.

### 3.2 Paternity Assignment

We assigned paternity to 72 seeds, coming from all 13 sampled mother plants. The assigned fathers were 48 individuals. For 11 seeds, the assigned father was also the mother, indicating self‐fertilization. Eight of the cases of self‐fertilization were detected in the same *M. longifolia* individual, whereas the other three cases occurred in three different individuals. For seeds from eight fruits, we assigned fathers to more than one seed coming from the same fruit. In six of these cases, the seeds had different fathers; in two cases, the seeds had the same father. A list of seeds and their assigned fathers can be found in the Appendix (Table A1).

### 3.3 Pollen Dispersal

**FIGURE 3:**
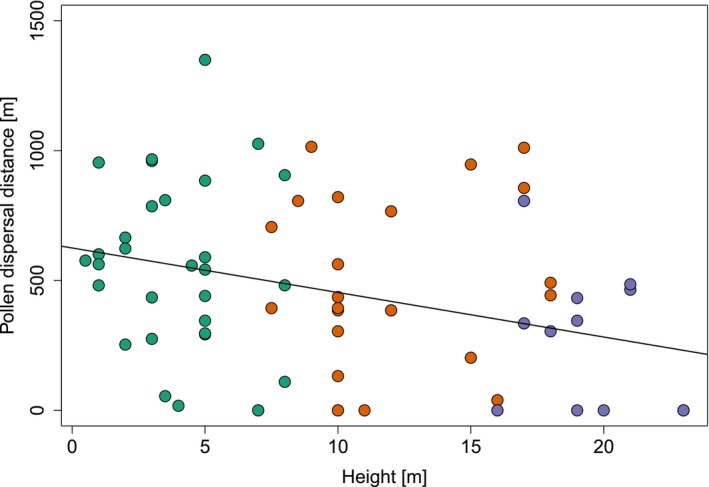
Relationship between pollen dispersal distance and the height of fruit collection for *M. longifolia* seeds (*n* = 72) at EBQB. The fitted linear model is: *Y* = 625.188–17.139 · x. The model indicates a significant negative relationship (multiple *R* ^2^ = 0.112, *p* = 0.004). Green dots represent the understory, orange dots the midstory, and purple dots the canopy.

**FIGURE 4:**
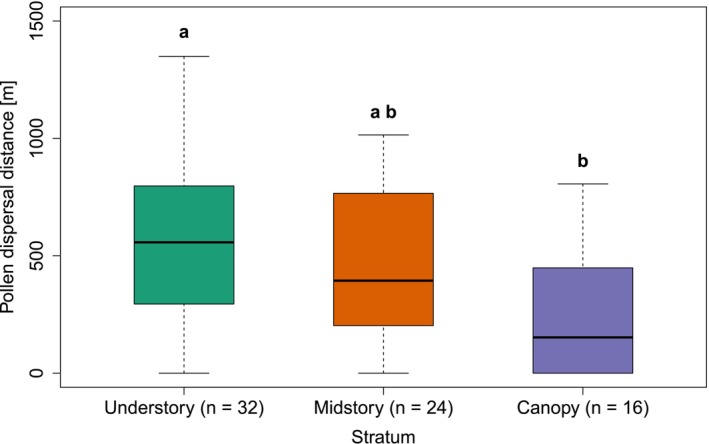
Pollen dispersal distances between strata for *M. longifolia* seeds (*n* = 72) at EBQB. Significant differences between strata are indicated by different letters (Kruskal‐Wallis test, *χ* ^2^ = 9.178, df = 2, *p* = 0.01). The boxes represent the interquartile range (IQR) for each stratum, with the horizontal line inside the box showing the median value. The whiskers extend from the box to the smallest and largest values within 1.5 times the IQR from Q1 and Q3.

Pollen dispersal distances ranged from 0 to 1350 m, with a median of 435 m (Q1 = 126 m, Q3 = 721 m). Pollen from flowers at lower heights was dispersed over larger distances (negative linear correlation, multiple *R* ^2^ = 0.112, *p* = 0.004; Figure 3). Accordingly, pollen dispersal distances were higher in the understory and midstory than in the canopy (Kruskal‐Wallis test, *χ* ^2^ = 9.178, df = 2, *p* = 0.01; Figure 4). The median pollen dispersal distance in the understory was 557 m (Q1 = 295 m, Q3 = 798 m), in the midstory 394 m (Q1 = 203 m, Q3 = 766 m), and in the canopy 152 m (Q1 = 0 m, Q3 = 440 m). Post hoc Dunn's test showed a significant difference between the understory and canopy (*Z* = 3.01, adjusted *p* = 0.01), whereas differences between the understory and midstory (*Z* = 0.90, adjusted *p* = 1.00) and between the midstory and canopy (*Z* = 2.15, adjusted *p* = 0.09) were not significant.

### 3.4 Spatial Genetic Structure (SGS)

**FIGURE 5:**
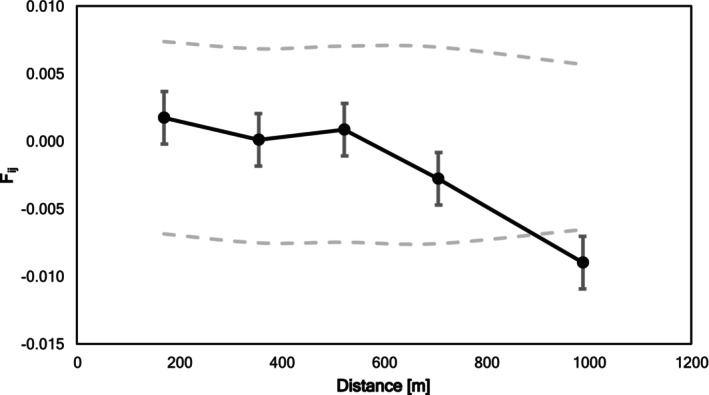
Average pairwise kinship over pairwise spatial distance [m] for adult *M. longifolia* individuals (*n* = 97). Pairwise kinship is determined by Loiselle's kinship coefficient (*F*_ij_). Dashed lines represent upper and lower 95% confidence intervals.

We did not detect a SGS for *M. longifolia* (Sp = 0.002 ± 0.004, *p* = 0.153). Estimates of SGS based on the Sp statistics for the first distance class were *F*_ij(1)_ = 0.0017, *b* = −0.002, and *r* ^2^ = 0.0002. Autocorrelation showed no signs of an SGS (Figure 5). Mean distances within distance classes ranged from 170 m for the first class to 988 m for the fifth class.

## 4 Discussion

We observed that pollen dispersal distances for *M. longifolia* varied widely, with longer distances generally occurring for pollen from flowers in lower forest strata. We were able to assign paternity to only a small fraction (21%) of the seeds included in our final dataset for paternity analysis. A major limitation was the need to discard half of our 12 initial genetic markers, leaving us with only six loci for genotyping and subsequent analyses. In many cases, not all six markers were successfully typed, and seeds with fewer than three loci genotyped were excluded, as this was insufficient for reliable paternity assignment. Ideally, we would have used 10 loci to confidently assign paternity to a greater proportion of seeds (Kalinowski et al. 2007); but this was not feasible due to challenges with our study species. Seeds of *M. longifolia* are extremely small and require significant time to germinate and grow, and often left us with very small amounts of leaf material for DNA extraction. This limitation likely affected both the quantity and quality of the extracted DNA. In contrast, DNA extraction from cambium samples of adult plants was much more successful, and we were able to genotype three or more loci for the vast majority of the adult *M. longifolia* individuals.

Although we showed that bats (specifically *H. thomasi* , *C. minor,* and *A. caudifer* (Thiel et al. 2024)) move pollen across the entirety of our study area, the sampling design limited the detection of longer‐distance pollen dispersal events, which occur when bats deliver pollen from donors outside the sampling range. Pollen dispersal distances by bats are highly influenced by environmental factors such as landscape structure and feeding behavior, which affect how far bats transport pollen (e.g., Diniz et al. 2019; Fleming et al. 2009; Horner et al. 1998; Klingbeil and Willig 2009). Collevatti et al. (2010) reported mean dispersal distances of 132 m for *Caryocar brasiliense* , a bat‐pollinated tree in the Brazilian Cerrado, while Biscaia de Lacerda et al. (2008) measured an average pollen flow distance of 827 m for the bat‐pollinated tree *Hymenaea courbaril* in the Brazilian Amazon. Maximum pollen dispersal distances depend on the maximum movement distances of the pollen‐dispersing bats, which are related to the home‐range size. Rothenwöhrer et al. (2011) documented home ranges of 12.5 ha for the small nectarivorous bat *Glossophaga commissarisi* in a Costa Rican lowland rainforest. Similarly, Heithaus et al. (1975) found a mean recapture distance of *Glossophaga soricina* of 370 m in a seasonal tropical forest in Costa Rica. These comparisons suggest that the pollen dispersal distances we observed for *M. longifolia* are relatively long, supporting the hypothesis that *H. thomasi* , *C. minor* , and *A. caudifer* move pollen across the entirety of our study area.

We detected higher pollen dispersal distances in the understory compared to the canopy. This finding aligns with our hypothesis, which was based on previous research investigating foraging visits by nectar‐feeding bats at different heights of *M. longifolia* (Thiel et al. 2024). If more bats visit inflorescences in the understory and midstory, likely, pollen dispersal distances in these strata would also be longer. However, the statistical power of our analyses was moderate due to the small sample size. We were able to assign paternity to more seeds from the understory and midstory than from the canopy, resulting in a relatively small number of canopy seeds with known fathers. This uneven distribution may limit the representativeness of our results regarding gene flow across vertical strata. The higher number of successfully assigned seeds in the lower strata corresponds to the larger number of seeds originally genotyped from these height categories. Thiel et al. (2024) documented bat visitation to *M. longifolia* flowers across the full vertical gradient, from the understory to the canopy. Bat species were identified only in the lower strata, as mist nets in that study reached up to 8 m, and species identification from camera trap footage was not feasible, so it remains unclear whether the same or different species are responsible for pollination across forest strata. We found no SGS in *M. longifolia* at our study site. This result supports our hypothesis, as we expected high gene flow due to the overall high mobility and diversity of pollen vectors (at least three bat species, Thiel et al. 2024) and seed dispersers (at least 41 bird species and two primate species, Thiel et al. 2023). Pollen and seed dispersal by these animals occurs over large distances, limiting the emergence of a SGS. The continuous habitat at our study site does not present any obvious barriers for bats, further supporting the absence of SGS. Long‐range dispersal by both pollen and seed vectors reduces the likelihood of SGS (Dick et al. 2008; Gelmi‐Candusso et al. 2019).

## 5 Conclusions

Through this research, we aimed to enhance our understanding of the ecological processes that drive gene flow in bat‐pollinated tropical plants, as well as how forest stratification influences genetic dynamics. Our study of the liana *M. longifolia* revealed that bats moved pollen over distances of up to 1350 m and that pollination distances were vertically stratified within the forest. These findings emphasize the significant role of bats as effective pollen dispersers, contributing to substantial gene flow across the study area. Moreover, given the scarcity of studies on the distance over which bats disperse pollen, our findings offer new insights into the ecological interactions that shape genetic connectivity in tropical rainforest ecosystems.

## Author Contributions

**Malika Gottstein:** data curation (equal), formal analysis (equal), investigation (equal), resources (equal), writing – original draft (lead), writing – review and editing (equal). **Sarina Thiel:** data curation (equal), investigation (lead), methodology (equal), resources (lead). **Jan Lukas Vornhagen:** data curation (equal), formal analysis (equal), investigation (equal), writing – original draft (supporting). **Christina Mengel:** data curation (lead), investigation (equal), methodology (equal). **Marco Tschapka:** conceptualization (equal), funding acquisition (equal), supervision (equal), writing – review and editing (equal). **Eckhard W. Heymann:** conceptualization (equal), funding acquisition (equal), supervision (equal), writing – review and editing (equal). **Katrin Heer:** conceptualization (lead), funding acquisition (equal), methodology (lead), project administration (lead), resources (equal), supervision (lead), writing – review and editing (equal).

## Conflicts of Interest

The authors declare no conflicts of interest.

## Data Availability

The *Marcgravia longifolia* data set, including type (adult or seed), ID of the known mother, seed ID, year of sample collection and genotyping, spatial reference, height class and genotypes for the microsatellites used in the analysis, is deposited on Zenodo (https://zenodo.org/records/14882215).

## Appendix A

**TABLE A1:** Seeds with successfully assigned fathers.

Seed ID	Mother	Assigned father
ML3_18_3_4_1	ML3	ML3
ML3_18_5_1_1	ML3	ML66
ML3_18_6_3_1	ML3	ML3
ML3_19_1_1_1	ML3	ML3
ML3_19_1_1_2	ML3	ML3
ML3_19_1_1_3	ML3	ML3
ML3_19_2_1_1	ML3	ML88
ML3_19_2_2_1	ML3	ML3
ML3_19_3_3_2	ML3	ML3
ML3_19_5_2_3	ML3	ML78
ML3_19_6_4_4	ML3	ML3
ML26_18_5_1_2	ML26	ML26
ML26_19_1_1_1	ML26	ML26
ML27_19_6_1_1	ML27	ML90
ML34_18_7_2_1	ML34	ML65
ML34_18_9_2_2	ML34	ML64
ML34_18_9_2_5	ML34	ML34
ML34_19_1_1_2	ML34	ML49
ML34_19_1_2_1	ML34	ML72
ML34_19_2_4_2	ML34	ML10
ML34_19_2_6_1	ML34	ML66
ML34_19_3_5_2	ML34	ML56
ML37_18_12_3_1	ML37	ML87
ML37_18_12_3_2	ML37	ML69
ML37_18_2_3_2	ML37	ML74
ML48_18_10_1_1	ML48	ML3
ML48_18_11_3_1	ML48	ML70
ML48_18_11_4_1	ML48	ML26
ML48_18_11_4_2	ML48	ML8
ML48_18_11_4_3	ML48	ML37
ML48_18_11_4_5	ML48	ML53
ML48_18_14_2_2	ML48	ML102
ML48_18_19_2_1	ML48	ML83
ML48_18_19_2_2	ML48	ML37
ML48_18_4_6_10	ML48	ML3
ML48_18_4_6_9	ML48	ML3
ML55_18_9_2_4	ML55	ML55
ML55_18_9_2_6	ML55	ML91
ML55_19_1_1_3	ML55	ML33
ML55_19_3_1_1	ML55	ML66
ML55_19_4_2_1	ML55	ML26
ML55_19_5_1_2	ML55	ML89
ML55_19_6_2_1	ML55	ML91
ML57_18_1_3_4	ML57	ML91
ML57_19_1_2_2	ML57	ML105
ML57_19_4_2_1	ML57	ML43
ML57_19_5_2_3	ML57	ML22
ML57_19_5_3_2	ML57	ML89
ML57_19_7_1_1	ML57	ML105
ML61_19_1_1_1	ML61	ML89
ML61_19_1_2_3	ML61	ML89
ML61_19_3_1_3	ML61	ML92
ML61_19_3_3_2	ML61	ML67
ML61_19_4_1_1	ML61	ML36
ML61_19_7_2_3	ML61	ML89
ML70_19_1_1_1	ML70	ML75
ML70_19_2_2_2	ML70	ML70
ML70_19_4_2_1	ML70	ML75
ML72_19_1_9_1	ML72	ML5
ML72_19_3_2_2	ML72	ML77
ML72_19_6_1_1	ML72	ML71
ML84_18_12_2_3	ML84	ML8

*Note:* Seed ID format: MotherID_Year_InfructescenceID_FruitID_SeedID. For example, the ID ML3_18_3_4_1 refers to seed 1 from fruit 4 of infructescence 3 collected from mother plant ML3 in 2018. Seeds from the same fruit only differ in the last number, seeds from the same infructescence differ in the last two numbers.
